# Depressive symptoms in early adolescence: the dynamic interplay between emotion regulation and affective flexibility

**DOI:** 10.3389/fpsyg.2024.1165995

**Published:** 2024-03-22

**Authors:** Brenda Volkaert, Laura Wante, Jan R. Wiersema, Caroline Braet

**Affiliations:** ^1^Department of Developmental, Personality and Social Psychology, Ghent University, Ghent, Belgium; ^2^Department of Experimental Clinical and Health Psychology, Ghent University, Ghent, Belgium

**Keywords:** affective flexibility, emotion regulation, young adolescents, depressive symptoms, computer task

## Abstract

Individual differences in affective flexibility may be an important factor in understanding how emotion regulation is associated with adolescents’ depressive symptoms. The current study explored the role of affective flexibility as a moderator in the relationship between the use of both adaptive and maladaptive emotion regulation strategies and depressive symptoms in 65 young adolescents (11–13 years). Affective flexibility was indicated by the reaction time and accuracy index on the Emotional Flexible Items Selection Task. Emotion regulation strategies and depressive symptoms were assessed by the use of self-report questionnaires. Contrary to the hypotheses, affective flexibility did not moderate the relationship between emotion regulation and adolescents’ depressive symptoms. Instead, only a direct effect of the use of adaptive emotion regulations strategies on adolescents’ depressive symptoms was found. Additionally, post-hoc analyses revealed that there may exist an indirect relationship between affective flexibility and adolescents’ depressive symptoms through the use of adaptive emotion regulation strategies. These findings help to formulate a better understanding of how emotion regulation and affective flexibility in early adolescents are related to depressive symptoms.

## Introduction

The period of adolescence is a critical developmental phase characterized by an increased risk for the development and maintenance of depressive symptoms, which may eventually lead to a full-blown depressive disorder (Thapar et al., 2012). Given the high prevalence and detrimental consequences associated with depressive symptoms in adolescents (Thapar et al., 2012), it is highly indicated to identify core mechanisms underlying their development and maintenance. A mechanism that received increasing attention in the depression literature is emotion regulation.

Gross (1998) defined emotion regulation as a set of strategies that individuals use to have an impact on which emotions are experienced, when they are experienced, and how they are experienced and expressed. In general, emotion regulation strategies can be categorized into adaptive and maladaptive strategies based on their association with the development and maintenance of psychopathology (Aldao et al., 2010, 2016). Adaptive emotion regulation strategies can be considered as protective factors for the development of psychopathology, as they successfully reduce negative affective states, strengthen or control positive affective states, and are known to restore emotional balance (Gross, 2013; Aldao et al., 2016). Maladaptive emotion regulation strategies can be considered as risk factors for the development of psychopathology, as they only offer short term relief, are unsuccessful in strengthening positive affect states, or fail to permanently reduce negative affect (Aldao et al., 2010). However, not only the use of adaptive vs. maladaptive emotion regulation skills is associated with better outcomes. Several researchers also highlighted the importance of flexibility when evaluating the adaptive nature of emotion regulation (e.g., Bonanno and Burton, 2013; Aldao et al., 2015). Flexible emotion regulation refers to the ability of an individual to select and apply different emotion regulation strategies in different situations depending on the contextual demands (Kobylińska and Kusev, 2019). Of note is that especially young adolescents temporarily fail to (flexibly) use adaptive strategies and are more likely to resort to (the rigid use of) maladaptive strategies, which increases their risk for developing psychopathology (Cracco et al., 2017).

There is strong evidence from cross-sectional and longitudinal studies that emotion regulation (strategies) may play an important role in the development and maintenance of depression (see Joormann and Stanton, 2016 for an overview). In general, symptoms of depression are related to an increased use of the so-called maladaptive emotion regulation strategies (e.g., rumination, avoidance, suppression), a decrease of the so-called adaptive emotion regulation strategies (e.g., distraction, cognitive reappraisal, acceptance), and less flexibility (Schäfer et al., 2017; Visted et al., 2018; Wen et al., 2021). Furthermore, Gonçalves et al. (2019) found that overall emotion regulation difficulties, and a limited access to emotion regulation strategies in particular, predict depressive symptoms from early to middle adolescence. However, it should be noted that emotion regulation strategies do not always show a (strong) association with depressive symptoms, indicating that there are third variables influencing this relationship.

One of the proposed variables underlying emotion regulation is the ability to attend and respond to goal-relevant information, while inhibiting attention and responses toward distracting information (i.e., cognitive control) (Braver, 2012). A handful of studies evidenced that the relationship between emotion regulation and depressive symptoms varies depending on individual differences in cognitive control processes (Pe et al., 2013; Southward and Cheavens, 2017; Godara et al., 2020). A particular cognitive control process that is highly valuable to discuss within an emotion regulation framework, is affective flexibility (Pruessner et al., 2020). Affective flexibility refers to the use of cognitive control in affective contexts and can be defined as the ability to focus attention on emotional stimuli, disengage attention from these stimuli, and shift flexibly between emotional stimuli in particular (Malooly et al., 2013; Schweizer et al., 2020a). Theory and adult research has already indicated that deficits in affective flexibility hinders adaptive emotion regulation and leads to a repetitive and overly rigid use of maladaptive emotion regulation strategies (Johnson, 2009; De Lissnyder et al., 2011; Malooly et al., 2013; Martins et al., 2018; Pruessner et al., 2020). Although studies in younger age groups are limited, Schweizer et al. (2020a) concluded that affective flexibilty can be considered as an important cognitive building block for adolescents’ effective emotion regulation and suggested its association with mental health problems. In line with this tought, Schweizer et al. (2020b) explored how affective flexibitliy was related to emotion regulation and mental health over the course of adolescence. Results showed that lower affective flexiblity was associated with higher difficulties in emotion regulation and more mental health problems, especially in early adolescence. Moreover, the findings indicated that deficits in affective flexibilty accounted for the variation in the relationship between adolescents’ emotion regulation and mental health, suggesting the moderating role of affective flexibility. Unfortunately, research investiging the dynamic interplay between affective flexibility and emotion regulation in the development of depressive symptoms is currently lacking.

Therefore, the main goal of this study is to examine whether the association between emotion regulation strategies and depressive symptoms is moderated by affective flexibility in a sample of young adolescents. We expect that the association between both adaptive and maladaptive emotion regulation strategies and depressive symptoms will be moderated by the level of one’s affective flexibility. More specifically, it is hypothesized that the negative relationship between adaptive emotion regulation strategies and depressive symptoms will be stronger for adolescents with high levels of affective flexibility, compared to adolescents with low levels of affective flexibility. As for the use of maladaptive emotion regulation strategies, we expect that the positive relationship with depressive symptoms will be stronger for adolescents with low levels of affective flexibility, compared to adolescents with high levels of affective flexibility.

## Method

### Participants

The current sample consists of 65 young adolescents (46% boys) aged between 11 and 13 years (*M =* 11.9, SD = 0.54), with 88% attending the regular school program and 12% attending an adjusted program for students with special needs. Regarding to socio-economic status, half of the participants (53%) lives in middle class families, 36% in high class families, and 11% in low class families. Data from seven participants was excluded from analysis due to drop-out (*N* = 2) or failure to comply with the instructions (*N* = 5). Two scores were excluded from analysis because they were higher than three standard deviations above the mean and therefore considered as univariate outliers. Thus, data of 56 participants was used to conduct the statistical analyses.

### Materials and procedure

Approximately 70 first-year students in the Flemish region of Belgium received information on the study and were invited to participate. Adolescents and their parents had to give active consent for participation and were free to withdraw from the study at any moment without any consequences. As five parents did not actively respond to the consent, 65 participants took part in the study. Each participant performed a 40-min computer task and filled out two self-report questionnaires. Before the adolescents started with the computer task, the researcher illustrated two trials from the computer task using printed paper cards. All measurements took place at school. The anonymity was guaranteed as adolescents received a unique code that they could use to log in to the computer task and register on the questionnaires. Study and data collection were approved by The Ethical Committee of the Faculty Psychology and Education Sciences of the Ghent University. After the study, participants were rewarded with a professional photoshoot with their classmates.

The Children’s Depression Inventory (CDI; Timbremont and Braet, 2002), a 27-item self-report questionnaire for children and adolescents between seven and 17 year old, was used to assess depressive symptoms. The CDI has demonstrated satisfactory psychometric properties in previous research (Timbremont and Braet, 2002) and showed good internal consistency in the current study with Cronbach’s *α* = 0.89.

The Fragebogen zur Erhebung der Emotionsregulation bei Kindern und Jugendlichen (FEEL-KJ, Braet et al., 2013), a 90-item self-report questionnaire for children between eight and 18 years old, was used to assess adolescents’ emotion regulation. Participants were asked to indicate how often they use specific adaptive, maladaptive, and external emotion regulation strategies in response to feelings of anger, sadness, and fear (see Table 1 for an overview). In the current study, only the overall scores on adaptive (Cronbach’s *α* = 0.70) and maladaptive emotion regulation strategies (Cronbach’s *α* = 0.97) were used. The FEEL-KJ has demonstrated a good reliability and validity (Braet et al., 2013).

**Table 1:** Emotion regulation strategies from the FEEL-KJ.

Emotion regulation strategies	Example of an item
Adaptive strategies	
Problem solving	I try to change what makes me angry
Distraction	I do something fun
Forgetting	I think it will pas
Acceptance	I accept what makes me angry
Humor enhancement	I think about things that make me happy
Cognitive problem solving	I think about what I can do
Revaluation	I tell myself it is nothing important
Maladaptive strategies	
Giving up	I do not want to do anything
Withdrawal	I do not want to see anyone
Rumination	I cannot get it out of my head
Self-devaluation	I blame myself
Aggressive Actions	I get into a quarrel with others
Other strategies	
Social support	I tell someone how I am doing
Expression	I express my anger
Emotional Control	I keep my feelings for myself

Based on Cracco et al. (2017).

**Figure 1:**
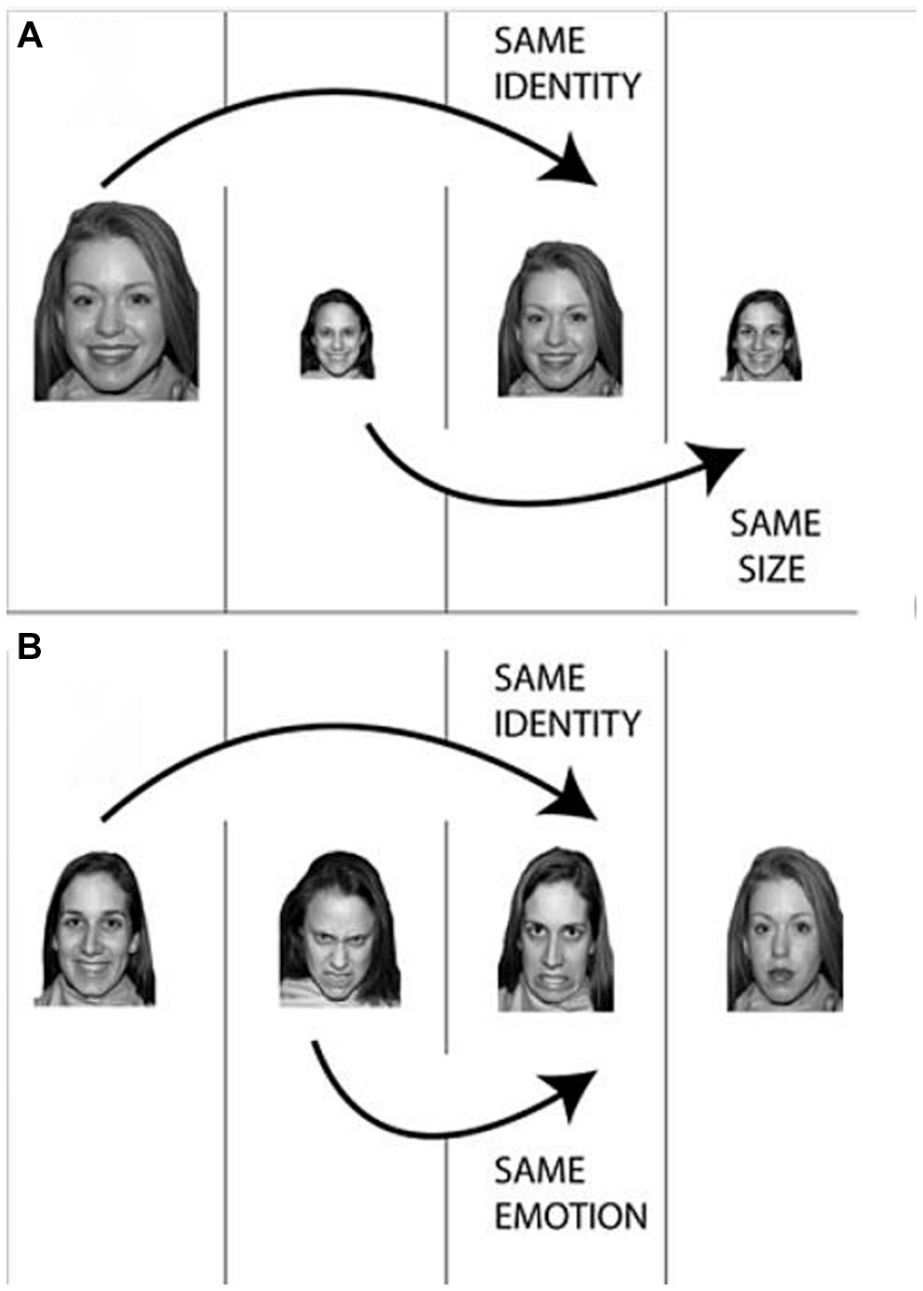
Description of the emotional flexible item selection task with **(A)** an example of a low difficulty trial and **(B)** an example of a high difficulty trial with a pivot stimulus. Reprinted with permission from Mărcus et al. (2016), Springer Nature.

The Emotional Flexible Items Selection Task (EM-FIST, Mărcus et al., 2016) was used to assess the level of affective flexibility. In the EM-FIST, participants see images of expressive faces that vary on three dimensions; identity (person 1, person 2, or person 3), size (small, medium, or big), and emotion (angry, happy, or neutral). The presented images are always equal to the gender of the participants. Per trial, four images that differ on two of the three dimensions are shown. The participant is asked to first identify a pair of images that is the same on one of the three dimensions, followed by identifying another pair of images that is equal on another dimension. In the low-difficulty trials, the identification of the two pairs does not involve a pivot stimulus (i.e., stimuli that needs to be identified in terms of two dimensions). In the high-difficulty trials, the identification of the two pairs involves a pivot stimulus, so participants need to shift between two stimuli in terms of dimension (see Mărcus et al., 2016 for a more detailed description). In Figure 1, an example of the two different trials is shown. The task includes two demonstration trials, which are guided by verbally prerecorded instructions, two practice trials, and 108 test trials, with an equal number of low and high difficulties trials that are presented in a randomized order. Each trial is presented for maximum 10 s and the inter-stimulus interval is 1 s. The level of affective flexibility is reflected in a reaction time index and an accuracy index, which are computed by subtracting the processing speed and accuracy of the low difficulty trials from the high difficulty trials. Participants with high affective flexibility show less performance differences between the two difficulty levels compared to participants with low affective flexibility. Participants completed the EM-FIST individually on a 16-inch laptop using E-Prime Software.

## Results

Variable means, standard deviations and non-parametric Spearman rank correlation coefficients are presented in Table 2. The use of adaptive emotion regulation strategies was significantly correlated with the use of maladaptive emotion regulation strategies, the accuracy index, and the depressive symptoms. Paired sample *t*-test showed that accuracy rates on the low difficulty trials (*M* = 0.78, *SD* = 0.23) were significantly higher compared to the high difficulty trials (*M* = 0.67, *SD* = 0.22, *t* (55) = −11.75, *p* < 0.001). Furthermore, the mean reaction time in the low difficulty trials (*M* = 1352.25, *SD* = 0.325.54) was significantly lower compared to the high difficulty trials [*M* = 1418.57, *SD* = 0.310.92, *t* (55) = 4.11, *p* < 0.001].

**Table 2:** Means, standard deviations and correlations of all variables.

Variable	*M*	SD	1	2	3	4
1. DEP	9.68	8.17	–			
2. AER	115.26	29.19	−0.60**	–		
3. MAER	41.64	10.22	0.24+	−0.35**	–	
4. ACC	−0.11	0.07	−0.18	0.30*	−0.20	–
5. RT	66.32	120.73	0.08	0.01	0.08	0.16

**p < 0.01, *p < 0.05, + *p* < 0.10; DEP, depressive symptoms; AER, adaptive emotion regulation strategies; MAER, maladaptive emotion regulation strategies; ACC, accuracy index, RT, reaction time index.

Four different moderation regression models were conducted with (1) the centered scores of the use of adaptive emotion regulation strategies or the use of maladaptive emotion regulation strategies, (2) the centered accuracy or reaction time index, and (3) the interaction effect between adaptive or maladaptive emotion regulation strategies and accuracy or reaction time index as independent variables, and depressive symptoms as dependent variable. To account for the constraints of null-hypothesis significance testing (see for example Jarosz and Wiley, 2014), Bayesian statistics were estimated for each interaction effect. Findings showed no significant interaction effects between the use of adaptive or maladaptive emotion regulation strategies and the reaction time index or the accuracy index. All Bayes Factors are less than 1, supporting the null hypothesis as being more likely than the alternative hypothesis. With regard to the main effects, the data yielded a significant effect of adaptive emotion regulation strategies on depressive symptoms, in such a way that adolescents who use less adaptive emotion regulation strategies reported a higher number of depressive symptoms and vice versa (see Table 3 for an overview).

**Table 3:** Linear models of predictors of depressive symptoms.

	*b*		*SE*	*t*	*p*	*BF*
Constant	9.88	[8.07, 1.68]	0.89	10.98	<0.01	
AER (centered)	−0.17	[−0.24, −0.11]	0.03	−5.58	< 0.01	
ACC (centered)	−1.78	[−27.03, 23.46]	12.58	−0.14	0.87	
AERxACC	−0.30	[−1.08, 0.48]	0.39	−0.76	0.45	0.154
*R*^2^ = 0.41						
Constant	9.68	[7.94, 11.41]	0.86	11.18	<0.01	
AER (centered)	−0.18	[−0.23, −0.12]	0.02	−7.15	< 0.01	
RT (centered)	−0.01	[−0.01, 0.01]	0.01	−0.04	0.97	
AERxRT	0.00	[−0.01, 0.01]	0.01	−0.10	0.92	0.105
*R*^2^ = 0.41						
Constant	9.92	[7.76, 2.08]	1.08	9.21	<0.01	
MAER (centered)	0.09	[−0.120; 0.309]	0.10	0.88	0.38	
ACC (centered)	−22.20	[−52.121; 7.720]	14.92	−1.49	0.14	
MAERxACC	2.30	[−0.752; 5.362]	1.52	1.51	0.13	0.307
*R*^2^ = 0.09						
Constant	9.65	[7.42, 11.89]	1.11	8.68	<0.01	
MAER (centered)	0.11	[−0.110; 0.341]	0.11	1.02	0.31	
RT (centered)	0.00	[−0.020, 0.017]	0.01	−0.16	0.87	
MAExRT	0.00	[−0.001, 0.001]	0.01	0.32	0.68	0.107

AER, adaptive emotion regulation strategies; MAER, maladaptive emotion regulation strategies; RT, reaction time index; ACC, accuracy index; BF, Bayes factor testing model versus null model.

The positive correlation between the accuracy index and the use of adaptive emotion regulation strategies, and the significant main effect of adaptive emotion regulation strategies on depressive symptoms, may indicate that the relationship between affective flexibility, adaptive emotion regulation, and adolescents’ depressive symptoms can be considered as a mediation model. This would be consistent with the study of Wante et al. (2017), which found evidence for the mediating role of emotion regulation in the relationship between cognitive processes and depressive symptoms among adolescents. As researchers already highlighted the need to distinct moderation from mediation models to explain depressive symptoms in adolescence (Grant et al., 2006), additional mediation analyses were conducted.

Null hypothesis Significance Testing showed no significant direct relationship between the accuracy index and depressive symptoms [*b* = − 25.10, 95% CI (−54.86, 4.67), *t* = −1.90, *p* = 0.10, *R^2^* = 0.05, BF = 0.413]. Instead, the data yielded a significant indirect effect of the accuracy index on depressive symptoms through the use of adaptive emotion regulation strategies [*b* = −22.95, 95 CI (−41.01, −5.61), BF > 100]. More specifically, the data showed that the accuracy index significantly predicts the use of adaptive emotion regulation strategies [*b* = 129.94, 95% CI (26.70, 233.18), *t* = 2.52, *p* = 0.01, *R*^2^ = 0.10, BF = 2.03], such that a higher accuracy index is related to a higher use of adaptive emotion regulation strategies (and vice versa). Next, the use of adaptive strategies significantly predicts the depressive symptoms [*b* = −0.17, 95% CI (−0.24, −0.11), *t* = −5.64, *p* < 0.001, *R*^2^ = 0.41, BF > 100], such that a higher use of adaptive emotion regulation strategies is associated with lower levels of depressive symptoms (and vice versa). As all Bayes Factors are higher than 1, they deliver anecdotal to extreme evidence for the mediation analyses.

## Discussion

Contrary to our expectations, we did not find support for the dynamic interplay between the use of adaptive or maladaptive emotion regulations strategies and affective flexibility in explaining depressive symptoms among young adolescents. Although this study was the first to explore the moderating role of affective flexibility, these results are in contrast with previous research providing evidence for the influence of other cognitive control processes on the relationship between emotion regulation and depressive symptoms (Pe et al., 2013; Southward and Cheavens, 2017; Godara et al., 2020). Worthy of note is that previous research focused mainly on the use of specific cognitive emotion regulation strategies, such as cognitive reappraisal or rumination, and less on behavioral strategies, such as distraction or avoidance. It is plausible, although speculative, that affective flexibility is more related to cognitive emotion regulation strategies than to behavioral emotion regulation strategies, and that the ability to be affectively flexible will become more important when considering depressive symptoms in relation with a specific cognitive strategy. In contrast with previous research (Schäfer et al., 2017), the current findings also lack evidence for a direct relationship between maladaptive emotion regulation and adolescents’ depressive symptoms. It is possible that in this age group, the (decreased) use of adaptive strategies may be first linked with depressive symptoms before maladaptive strategies are acquired (Braet et al., 2014).

Inspired by prior research (e.g., Wante et al., 2017), and based on the observed correlations between the three variables of interest, we decided to run a *post hoc* mediation analysis. The data showed preliminary evidence for the fact that adolescents’ ability to be affectively flexible is associated with depressive symptoms, in such a way that adolescents with lower levels of affective flexibility reported more depressive symptoms, compared to adolescents with higher levels of affective flexibility. This finding is in line with multiple studies that have examined and supported the role of (affective) inflexibility and depression in adults (see Stange et al., 2017 for an overview). In general, it is suggested that a poor affective flexibility is associated with depression as it prolongs the processing of negative information, and leads to the exacerbation and preservation of negative affect. Although studies examining this specific relationship in adolescence are non-existent, our findings are consistent with the majority of the related literature, which has identified general cognitive control deficits in adolescent depression (see for example Holler et al., 2014).

Moreover, the effect of adolescents’ ability to be affectively flexible on depressive symptoms, seems to be fully mediated by the use of adaptive emotion regulation strategies. The current findings may be explained by the fact that the capacity to flexibly process emotional material (i.e., affective flexibility) is a prerequisite for the use of specific emotion regulation strategies, such as distraction (Johnson, 2009) and cognitive reappraisal (Malooly et al., 2013), which may be considered as adaptive under certain contexts. Moreover, in light of the more recent insights on emotion regulation flexibility, one can argue that a poor affective flexibility (also) complicates the ability to alternate between different strategies or to select the best strategy according to a specific situation, leading to a rigid strategy use (Aldao et al., 2015).

Despite the novelty and strengths of the current study, some limitations have to be acknowledged. The first limitation is related to the sample of the study. As the mean of the depressive symptoms scores was relatively low (*M* = 9.52), and the majority (80%) of the participants did not reached the threshold for clinical depression, results cannot be generalized to a clinical population. Furthermore, the current study sample is limited by a relatively small age range (11–13 years). As emotion regulation strategies and cognitive control processes such as affective flexibility are still under development (Cracco et al., 2017), the influences of these variables can depend on the developmental phase they are in. Finally, it is possible that the relatively small sample size may have deceased the probability to detect interaction effects. *Post hoc* power analyses revealed that the current analyses had 70% probability of correctly detecting an effect, which is lower compared to the power that is generally considered as adequate (80%) (Sedgwick, 2013). In addition, it is possible that characteristics such as age, gender, education, and socioeconomic status, have affected the results. However, due to the lack of power these possibilities were left unexplored. Research in a larger sample, a clinical sample, and in a sample of mid-through late adolescents is needed to address this question and make generalization possible.

Second, the study lacks the assessment of cognitive flexibility next to affective flexibility, which is needed to clarify whether the effect of flexibility is specific for processing emotion material or also applies for non-emotional material [see for example Martins et al. (2018)]. In addition, further research should also include the assessment of anxiety symptoms as there is a high comorbidity between symptoms of anxiety and depression in adolescents. Furthermore, Mărcus et al. (2016) showed that adolescents high in trait anxiety accomplish the high difficulty trials of the EM-FIST with higher reaction times compared to adolescents with low trait anxiety. Including anxiety could elucidate whether the indirect effect of affective flexibility through the use of adaptive emotion regulation strategies is depression-specific or not.

Third, given that emotion regulation and depressive symptoms were measured by the use of self-report questionnaires, it is possible that the obtained results are biased by an incorrect understanding of the questions, the tendency to give socially acceptable answers, and a decreased variability between individuals. Especially for emotion regulation, results may be biased by an individuals’ confidence or belief in their ability to successful modify emotions, rather than their actual (objective) emotion regulation skills and strategy use (Salters-Pedneault et al., 2006). We emphasize that future research needs to consider a multi-method and multi-informant approach and use self-report questionnaires in combination with other assessment techniques. In addition, Brieant et al. (2018) stated that the moderating effect of cognitive control processes such as affective flexibility may not be observable at a behavioral level, and therefore may be missed during computer tasks. Therefore, the additional use of neuroimaging research could shed a new light on findings. A final limitation is that the current study is limited by a cross-sectional design, which make it impossible to make causal or temporal inferences based on the current findings. It is possible that emotion regulation, whether or not in interaction with affective flexibility, indeed precedes depressive symptoms. However, it is also possible that both are caused by depressive symptoms. Longitudinal studies are needed to further examine these possibilities.

## Data availability statement

The raw data supporting the conclusions of this article will be made available by the authors upon reasonable request.

## Ethics statement

The studies involving humans were approved by the Faculty of Psychology and Educational Sciences Ethics Committee (Ghent University). The studies were conducted in accordance with the local legislation and institutional requirements. Written informed consent for participation in this study was provided by the participants’ legal guardians/next of kin.

## Author contributions

BV, LW, JW, and CB designed the study and devised the main conceptual ideas, BV and LW performed the experiments, BV processed the experimental data, performed the analysis, drafted the manuscript, and designed tables and figures. All authors discussed the results, provided critical feedback, and helped shape the research, analyses and manuscript. All authors approved the current version of the manuscript to be published.
